# Improved Sample Selection and Preparation Methods for Sampling Plans Used to Facilitate Rapid and Reliable Estimation of Aflatoxin in Chicken Feed

**DOI:** 10.3390/toxins13030216

**Published:** 2021-03-16

**Authors:** James Kibugu, Raymond Mdachi, Leonard Munga, David Mburu, Thomas Whitaker, Thu P. Huynh, Delia Grace, Johanna F. Lindahl

**Affiliations:** 1 Biotechnology Research Institute, Kenya Agricultural and Livestock Research Organization, P.O. Box 362, Kikuyu 00902, Kenya; rayelliemdachi@gmail.com; 2 Department of Biochemistry, Microbiology and Biotechnology, School of Pure and Applied Sciences, Kenyatta University, P.O. Box 43844, Nairobi 00100, Kenya; nmburu01@gmail.com; 3 Department of Animal Science, School of Agriculture and Enterprise Development, Kenyatta University, P.O. Box 43844, Nairobi 00100, Kenya; munga.leonard@ku.ac.ke; 4 Department of Biological and Agricultural Engineering, North Carolina State University, Box 7625, Raleigh, NC 27695-7625, USA; whitaker@ncsu.edu; 5 Hygiena LLC, Santa Ana, CA 92704-6804, USA; thuynh@hygiena.com; 6 Department of Biosciences, International Livestock Research Institute, P.O. Box 30709, Nairobi 00100, Kenya; D.Randolph@cgiar.org (D.G.); J.Lindahl@cgiar.org (J.F.L.); 7 Department of Clinical Sciences, Swedish University of Agricultural Sciences, 75007 Uppsala, Sweden; 8 Department of Medical Biochemistry and Microbiology, Uppsala University, 75123 Uppsala, Sweden

**Keywords:** aflatoxin, chicken feed, representative sampling, improved aflatoxin test procedure, validation

## Abstract

Aflatoxin B1 (AFB1), a toxic fungal metabolite associated with human and animal diseases, is a natural contaminant encountered in agricultural commodities, food and feed. Heterogeneity of AFB1 makes risk estimation a challenge. To overcome this, novel sample selection, preparation and extraction steps were designed for representative sampling of chicken feed. Accuracy, precision, limits of detection and quantification, linearity, robustness and ruggedness were used as performance criteria to validate this modification and Horwitz function for evaluating precision. A modified sampling protocol that ensured representativeness is documented, including sample selection, sampling tools, random procedures, minimum size of field-collected aggregate samples (primary sampling), procedures for mass reduction to 2 kg laboratory (secondary sampling), 25 g test portion (tertiary sampling) and 1.3 g analytical samples (quaternary sampling). The improved coning and quartering procedure described herein (for secondary and tertiary sampling) has acceptable precision, with a Horwitz ratio (HorRat = 0.3) suitable for splitting of 25 g feed aliquots from laboratory samples (tertiary sampling). The water slurring innovation (quaternary sampling) increased aflatoxin extraction efficiency to 95.1% through reduction of both bias (−4.95) and variability of recovery (1.2–1.4) and improved both intra-laboratory precision (HorRat = 1.2–1.5) and within-laboratory reproducibility (HorRat = 0.9–1.3). Optimal extraction conditions are documented. The improved procedure showed satisfactory performance, good field applicability and reduced sample analysis turnaround time.

## 1. Introduction

Aflatoxins are food-borne toxins produced by *Aspergillu*s fungi sections *Flavi*, *Ochraceorosei* and *Nidulantes*. Some aflatoxin producing species are *A. flavus*, *A. parasiticus*, *A. nomius, A. minisclerotigenes* and *A. arachidicola* whose aflatoxigenic strains are widespread in agricultural commodities, food and feed [1,2]. There are four types designated as aflatoxin B1, B2, G1 and G2 [3], found as natural dietary contaminants [4,5]. Metabolites such as aflatoxin M are found in edible animal products [5]. Aflatoxin B1 (AFB1), the most toxic and prevalent [2], is a potent human carcinogen [6]. Aflatoxins are moderately stable under normal cooking and industrial processing procedures [4,7,8]. There have been reports of acute human and animal aflatoxicosis outbreaks resulting in deaths [9,10] and widespread exposure to chronic dietary aflatoxins [2,11]. Prevalence data of aflatoxins contamination in poultry feeds are scanty particularly in low- and middle-income countries and characterized by wide variation. Levels of 36 ppb (mean) aflatoxin B1 was observed in Sudan [12], 100 ppb (mean) in India [13], 10–166 ppb aflatoxin in Pakistan [14], 74 ppb (mean) in Nigeria [15], 2.7 ppb (median) in Argentina [16] and 20–50 ppb in Kenya [17]. Recently, aflatoxin levels of 7.5–393.5 ppb in feed processing plants samples and 19.0–188.5 ppb in samples collected from farmers in Uganda [18] and 0.2–318 ppb in 2020 in Kenya were reported [19]. Maximum allowable limits for aflatoxin content in human food and animal feed have been established in more than 100 countries [20]. For total aflatoxins, the United States set a maximum guidance level of 20–300 ppb in animal feed and 20 ppb in human food [21], while it is 4 ppb in human food as set by the European Union (EU) [22]. Other than for dairy feed, AFB1 residues in animal feed are not usually regulated [20]. Uniquely, however, the EU has established a threshold for this mycotoxin in several animal feed matrices [23].

Chronic aflatoxicosis aggravates disease pathogenesis, impairs animal nutrition and productivity [6,24,25]. Aflatoxins are also teratogenic, carcinogenic, mutagenic, estrogenic, nephrotoxic, hepatotoxic and immunosuppressive [2,6,26,27,28]. Aflatoxins promote development of human primary hepatocellular carcinoma through synergy with the hepatitis B virus and has been associated with childhood stunting [29]. In chicken, dietary aflatoxins decrease feed intake and productivity and impair reproduction, causing economic losses, increased susceptibility to disease, poor vaccine response and toxin residues in poultry products [4,11,30]. In fact, dietary aflatoxin can reduce weight gain by 11% and increase mortality by 2.8% in chicken [31]. Aflatoxin contamination also causes food insecurity and economic impact through its adverse effect on international trade [8]. Dietary aflatoxin is therefore a public health concern of paramount importance that requires accurate estimation to enable employment of appropriate intervention strategies. Substantial efforts have been made to improve sensitivity and throughput of the analytical methods used for estimation of aflatoxin in food and feed. Thus, great achievements have been accomplished in the improvement of the analytical characteristics of the instrumental detection methods, e.g., liquid chromatography tandem mass spectrometry [4]. Nonetheless, for detection of trace levels of target analytes such as dietary aflatoxins, it is equally important that sample collection and preparation procedures are also optimized for accurate and rapid determination of the mycotoxin content.

Aflatoxin detection methods include immunoassays [16,32], fluorimetry [18] and chromatographic methods [14,32,33,34] such as LC-MS/MS for multi-mycotoxin analysis [15,19]. While these methods have different performance, the largest uncertainty associated with the measurement of aflatoxin content is due to lack of homogeneity of the contaminant in food and feed leading to variability [4,35]. It is indeed not easy to get a representative sample [11,36,37] that accurately estimates true aflatoxin content in a bulk consignment, as observed by Matumba et al. [38]. Another source of measurement uncertainty is bias, deviation from the true value due to sampling tools [39]. Aflatoxin analysis in food and feed is a three-step process: selection of the sample of a given size, sample preparation and quantification [4,40]. Development of chemical analysis often focuses on the last step, yet the sample selection step is the largest source of variability, followed by sample preparation, while quantification is the smallest contributor [35,37,39,41]. High variability necessitates increase in replicates to achieve required accuracy, thereby increasing the sample analysis turnaround time. There is need for test procedures with improved accuracy and precision for estimation of true aflatoxin exposure to ensure feed safety [36]. Recent data on aflatoxin contamination in figs [35] and maize [42] show that optimization of upstream procedures can considerably reduce the measurement uncertainty. In this study, sample selection, reduction and extraction steps were designed and validated to ensure representativeness of collected samples as well as appropriateness of the procedures and sampling tools used for estimation of aflatoxin residues in chicken feed. We first optimized sample selection procedures, and then incorporated a wet milling (water slurring) step in the feed sample preparation procedure, a critical modification that enhances sample homogenization more effectively than dry milling and a lesson learned from food analysis [35,39,41,42,43]. This reduces inter-assay variability and need for measurement replications thus decreasing sample analysis turnaround time. Because of lack of national, regional and international legal regulatory limits for AFB1 content in chicken feed, the EU legal framework was used as a reference in this study.

## 2. Results and Discussion

### 2.1. Highlights of Major Modification of the Improved Aflatoxin Test Procedure

The main modification in the five segments of the aflatoxin test procedure are as follows:

(a) Primary sampling or sample selection (number and size of incremental samples, type of sampling tools for open and closed sub-lots, random procedure, size of incremental and aggregate samples, Section 4.1.1)
(b) Secondary sampling (size of laboratory sample determined employing FAO Mycotoxin Sampling Tool, coning and quartering method improved by performing all coning and shoveling procedures under a steadfast funnel for mass-reduction of aggregate sample to 2 kg laboratory sample, Section 4.1.2)
(c) Tertiary sampling (size of test portion determined employing FAO Mycotoxin Sampling Tool, the improved coning and quartering method in Point (b) for mass-reduction of laboratory sample to 25 g test portion, Section 4.1.3)
(d) Quaternary sampling (homogenization and splitting of test portion by water slurring at matrix/water, 25:37.5, *w*/*w*; optimal matrix to organic solvent ratio for solid–liquid extraction, slurry/extraction solvent, 1.3:86.5, *w*/*v*, Section 4.1.4)
(e) Quantification of AFB1 (optimal organic solvent to aqueous buffer ratio for AFB1 extraction back to aqueous phase-modified extract to aqueous buffer mixture was modified to 80% acetonitrile extraction solvent: PBS-T mixture, 100:650, *v*/*v*, Section 4.1.5)

### 2.2. Enzyme-Linked Immunosorbent Assay of Prepared Standards for Determination of Aflatoxin Content in Chicken Feed Samples

**Figure 1:**
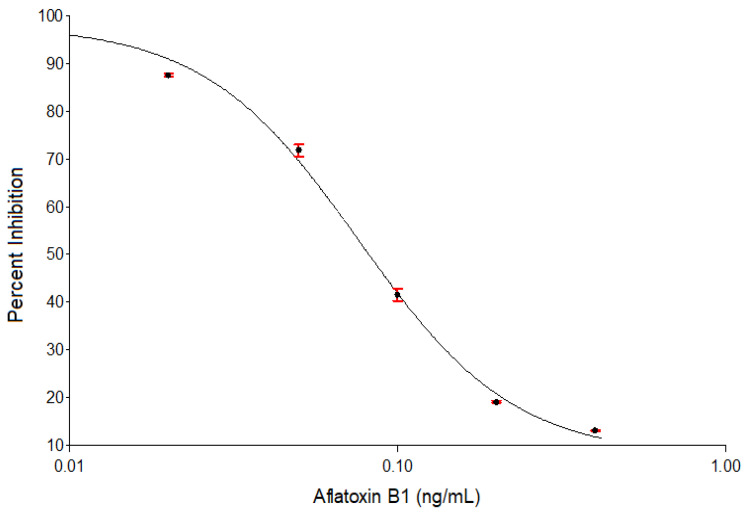
Four-parameter logistic standard curve of duplicate analysis of aflatoxin B1 standards in modified acetonitrile extract solvent: phosphate buffered saline-20 mixture (100:650, *v*/*v*). Extraction of aflatoxin B1 from organic into aqueous phase using the modified extraction solvent mixture did not affect the assay performance since the percent inhibition values were within the range specified by the manufacturer.

Aflatoxin B1 cELISA, using AFB1-ELISA low matrix kit (Helica Biosystems Inc.^®^, Santa Ana, CA, USA) is the last segment of the improved aflatoxin test procedure (Section 4.1) and its details are in Section 4.1.5. Curve-fitting characteristics of this immunoassay are shown in Figure 1. The four-parameter logistic curve (4PLC) of spiked aflatoxin B1 (AFB1) concentrations was characterized by two plateau regions and an inflection point (Figure 1). This curve was used to study linearity of measurements of AFB1 spiked in the modified extract to aqueous buffer mixture, 80% acetonitrile extraction solvent: PBS-T mixture (100:650, *v*/*v*).

Response (inhibition), y = a − d/[1 + (x/c)b] + d(1)

where x = AFB1 concentration and a–d are described in Table 1.

Numerous methods have been developed for analysis of aflatoxins in food and feed with high performance liquid chromatography (HPLC) as the gold standard. However, enzyme-linked immunosorbent assay (ELISA) has also been widely used given its many advantages over HPLC and other chromatographic techniques. ELISA is cost-effective because it does not need clean-up columns and expensive instrumentation, user-friendly, high-throughput, accurate and reproducible. One disadvantage of ELISAs is susceptibility to matrix effects, causing the reported analyte level to be falsely elevated or depressed. We used an ELISA method designed to be resistant to matrix interferences and which was previously validated to test disparate sample types, such as pet food [44], sorghum [45], maize [46], nuts, spices and many others [47]. Lack of requirement for sample clean-up and specialized equipment, skilled personnel for quantification by ELISA together with improved upstream sample handling procedures described herein, combined with high throughput (42 samples per run), guarantee rapid and reliable estimation of aflatoxin in complex and amorphous matrices such as animal feed. In addition, incorporation of a water slurring step in the extraction procedure reduces the number of test replications, considerably reducing sample analysis turnaround time.

### 2.3. Validation Results of the Improved Aflatoxin Test Procedure

Details of experimental design used for method validation are given in Section 4.2.2. Fourteen groups of native pseudo blank feed aliquots were used for method validation (Table 2). Briefly, Groups I–V were used to evaluate extraction efficiency and repeatability studies of surrogate aflatoxin (spiked analyte), Groups IV–XI were used for replication studies (within laboratory repeatability and reproducibility) of native aflatoxin (naturally occurring analyte) and Groups XII–XIV were used to evaluate limits of detection (LOD) and quantification (LOQ).

We modified primary sampling procedures to improve representativeness and ensure sample integrity. Uncertainty associated with sample selection was minimized through careful calculation of incremental and laboratory samples’ size using granulometry and particle size of matrix, together with increased number and size of test portions [39,40], correct design of sampling equipment to eliminate bias and random sampling procedures [48,49]. Specifically, the incremental samples were optimally spaced [48]. Sample size, sample selection and handling are usually dismissed as a “simple procedure”, but they are major source of variation. Attention should be given to the sample selection process for a given sample size as described herein.

#### 2.3.1. Method Accuracy and Precision

Extraction efficiency and variability associated with various aflatoxin extraction procedures for recovery studies are shown in Table 3 and raw data in Supplementary Materials (Dataset S1. Aflatoxin recovery data). Wet milling method (Group II) had mean aflatoxin recovery of 121%, which at *p* = 0.05 is not significantly different from mean recovery of 80% of conventional dry milling procedure (Group I). For estimation of spiked aflatoxin, variability associated with dry milling was significantly higher compared to wet milling. Coefficient of variation (CV) also referred to as relative standard deviation (RSD) of dry milling procedure (Group I) was 2.3-fold compared to slurry, wet milling method (Group II). CV effect associated with Group II (wet milling procedure) was less (CV rank 2) compared to CV rank 4 of Group I (dry procedure) (Table 3). Multiple comparisons employing Welch ANOVA-associated Games–Howell post-hoc test showed that mean aflatoxin recovery associated with Group III procedure was significantly (*p* < 0.05) different from those of both Groups IV and V (Table 3). Of the three wet milling methods, variability (Observed CV) associated with Group V (CV rank 1) was remarkably low compared to Groups III (1.5-fold) and IV (3-fold) procedures. Additionally, Group V had the least bias and repeatability precision (HorRat value < 1). Wet milling procedure associated with Groups II and V had acceptable precision level (expressed as CV or RSD) as prescribed by modified Horwitz equation. Analyzed together, the extraction procedure associated with Groups II and V had the lowest percent bias of −4.95, acceptable variability and recovery (Table 3: HorRat value < 2; recovery = 95%).

Precision (variability) was evaluated using the Horwitz equation where the measure of variation, predicted relative standard deviation (RSD_p_) or CV is a function of the analyte concentration [50,51,52,53,54]. This is given by modified and unmodified Horwitz equations:

For modified Horwitz equation, RSD_p_ < 2 ^(1−0.5logC)^ × 0.67 (2)

For unmodified Horwitz equation, RSD_p_ < 2 ^(1−0.5logC)^ (3)

where C = AFB1 concentration.

The modified Horwitz equation was used to predict RSD under repeatability and routine inter-assay conditions while the unmodified form was used for within-laboratory reproducibility conditions (intermediate precision). The observed relative standard deviation (RSD_O_) was compared with the RSD_p_ to give a Horwitz Ratio (HorRat) value, thus:

HorRat value = RSD_O_/RSD_p_ (4)

During method validation, the Commission of the European Communities (CEC) precision requirement for both repeatability and within-laboratory reproducibility conditions is a HorRat value < 2 [55]. However, for routine work (Section 2.3.5 and Section 4.2.8), we adopted inter-assay precision level of HorRat ≤ 1 [56].

Variability associated with estimation of natural AFB1 in chicken feed employing two sample splitting techniques at two sampling stages are shown in Table 4 and raw data in supplementary material (Dataset S2. Precision data). HorRat values for repeatability (HorRat**_r_**) and within-laboratory reproducibility (HorRat**_R_**) ranged 0.3–5.8 and 0.9–1.3, respectively, with all groups but one having HorRat values below the maximum allowable limit of 2 set by European legislation [55]. The lowest intra-laboratory variation was observed in secondary sampling for splitting 25 g dry aliquots from the laboratory sample (Group VI; RSD**_r_**= 6.5%, HorRat**_r_** value = 0.3; rank H1), and highest for preparing 1.3 g dry aliquots at tertiary sampling employing modified coning and quartering procedure (Group VIII; RSD**_r_**= 91.5%, HorRat**_r_** value = 5.8; rank H6). For preparation of 1.3 g analytical samples, intra-laboratory variation associated with coning and quartering procedure (Group VIII) was 4.4-fold that for water slurry (wet milling) method (Group IX, RSD**_r_**= 20.6%, rank H3). At tertiary sampling stage, intermediate precision (within-laboratory reproducibility) associated with Group X (RSD**_R_**= 30.3%, HorRat**_R_** value = 1.3, rank H4) and Group XI (RSD**_R_**= 26.9%, HorRat**_R_** value = 0.9, rank H2) of water slurry procedure were almost the same (HorRat value > 2), the latter having slightly lower variability. One-way ANOVA showed no significant effect (*p* > 0.05) of the analyst or the day of analysis on means of AFB1 levels for each condition of the two water slurry procedures.

The second most important source of variability after sample collection is the sample preparation segment of the aflatoxin test procedure [41,48]. Variability associated with splitting 25 g test portions from the comminuted laboratory sample employing modified coning and quartering procedure was much below the threshold level prescribed by European Union [52] and therefore suitable mass reduction method for this purpose. Density effects and matrix particle size influence performance of sample splitting methods [57]. We minimized this by efficient dry comminution of the aggregate sample prior to mass reduction [58]. Preparation of smaller size aliquots did not yield desirable precision under intra-laboratory conditions. Indeed, the FAO sampling tool [59] will not accept mass reduction in granular products beyond paired 25 g test portions for aflatoxin analysis because this will compromise representativeness. To enhance sampling precision, aggregate sample collected should not be less than 2 kg. Variability at this level can be reduced by increasing aggregate sample size before comminution through collection of 200 g incremental samples from all potential sampling locations. Removal of test portions larger than 25 g from a 2 kg (or larger) comminuted laboratory sample will reduce variability and can still be water slurried and a small (1.3 g) slurry aliquot selected for extraction. There are also commercial laboratory mills that incorporate a sample-splitting mechanism [4,41]. However, these are expensive and not readily available. Our novel aflatoxin test procedure is designed especially for laboratories that do not have automated sample splitting facilities.

Another critical innovation described here is inclusion of wet milling (water slurring), an additional comminuting step in the extraction procedure followed by processing of a smaller slurry aliquot. This allowed analysis of an adequately large test portion (25 g and larger), minimizing huge sampling uncertainty associated with aflatoxin estimation in animal feed and with reduced extraction cost. As reported in the literature, aflatoxin contamination is characterized by heterogeneous spatial distribution and nugget effect [37,60]. Wet milling is more efficient than dry milling in producing a more homogenous sample [35,39,43,61]. Indeed, water slurring was recently incorporated as a sample homogenizing procedure for aflatoxin analysis in maize [42] and dried figs [35]. This is the first report of using wet milling sample preparation method for aflatoxin analysis in animal feed. Through optimization of sample selection and mass reduction procedures and minimizing spatial heterogeneity of aflatoxin distribution in the test portion by wet milling, we were able to reasonably reduce measurement uncertainty and extraction cost. However, our modification does not completely eradicate inherent variability associated with sample selection, sample preparation and analytical steps of the aflatoxin test procedure, but minimizes this variability at each step, as well as reducing bias at both test portion selection and analytical segment. The aflatoxin diagnosis kit used in this study was not validated specifically for chicken feed. It is designed for various food matrices and animal feed grouped as one matrix. Because animal feed is an amorphous matrix with diverse physicochemical properties which can be a source of variation due to within-class matrix effects [62], we generated validation data associated with chicken feed. Single-laboratory validation of the modified aflatoxin test procedure was carried out through collection of data on spiking and recovery, replication, LOD and LOQ, robustness and ruggedness as the CEC prescribes [50,51,52,54,63].

Accuracy is a trade-off between bias and recovery data variability. We observed least bias (−4.95) and good aflatoxin recovery (95.1%) in wet milling procedure (Group. V). Recovery, precision and efficiency were compliant with CEC requirements of 75–125% for recovery and HorRat < 2 for precision [52,54,64]. AOAC and other authorities also recognize HorRat < 2 as a reliable precision criterion [64,65,66]. For replication studies using native aflatoxin, precision data collected under repeatability and reproducibility conditions met the EU guidelines. In absence of collaborative trial data, we estimated inter-laboratory precision using modified Horwitz equation against our within-laboratory reproducibility data, an internationally accepted practice [62]. Repeatability was both within the EU requirements and the same range for surrogate and native aflatoxin contents. Since native aflatoxin contamination is characterized by heterogeneity [4,35], we attribute the observed reduced variability to effective sample homogenization through the wet milling innovative procedure in our novel method described herein. By CEC requirements, the wet milling method described here has good repeatability and reproducibility. Reducing intrinsic variability associated with aflatoxin heterogeneity is cost effective in terms of time and resources [67].

#### 2.3.2. Limits of Detection (LOD) and Quantification (LOQ) Values

LOD and LOQ values associated with different sample preparation procedures are shown in Table 5 and raw data in Supplementary Materials (Dataset S3. LOD and LOQ data). The wet milling (slurry) procedure exhibited the strongest and most stable signals with the highest signal-to-noise ratio. These conditions yielded the lowest LOD (7.5 ppb) and the only value below the EU legal limit for AFB1 residues in animal feed of 10 ppb [23]. LOD and LOQ values associated with the slurry procedure were 7.5 and 16.0 ppb, respectively.

According to EU requirement, the maximum allowable AFB1 content in complete feed is 50 ppb (adult cattle, sheep and goats), 20 ppb (adult pigs and poultry) and 10 ppb (young animals) [23]. The LOD of our method is 7.5 ppb, well below the lowest regulatory limit. Other quantification methods can be incorporated with an obvious advantage of lowering LOD if so desired. Alternatively, levels below LOD can be estimated by extrapolation using a company program [68], substituting the values with the LOD divided by the square root of two [69] or replacing by half the LOD value if the below LOD results are less than 60% of the data [70,71], the latter being an approach used by several workers [72,73,74,75].

#### 2.3.3. Linearity

Expected and observed AFB1 concentration values of the prepared standards are shown in Table 6. There was significant (*p* < 0.01) negative correlation between ODs and measured aflatoxin levels of the prepared standards (Pearson correlation coefficient, r = −0.932). The expected and observed aflatoxin levels of the standards had a significant (*p* < 0.01) positive correlation (Pearson correlation coefficient, r = 0.961). The linear response range of the assay is 0.02–0.4 ng/mL, beyond which linearity was lost (Table 6). These results and curve-fitting characteristics (Figure 1) indicate that the modified sample preparation conditions did not affect the assay range. Values of coefficients of correlation and determination (Section 2.2) showed good linearity of measured inhibition and AFB1 concentration in the range of 0.02–0.4 ng/mL.

#### 2.3.4. Robustness and Ruggedness of the Aflatoxin Extraction Procedure

For robustness data, Groups II and V (slurry extraction method at final dilution factor of 1247) had HorRat values of less than 2 (Table 3), indicating stable extraction efficiency precision of the improved aflatoxin test procedure under intra-laboratory repeatability conditions suggesting more satisfactory robustness compared to the other extraction conditions (see raw data at Dataset S1. Aflatoxin recovery data of supplementary files). For ruggedness results, HorRat value for Group X (final dilution factor = 2500) was greater than 1 but less than 2, while, for Group XI (final dilution factor = 1247), it was less than 1 (Table 4), indicating more stable precision under reproducibility conditions for Group XI (see raw data at Dataset S2. Precision data of supplementary files). This suggests that the improved aflatoxin test procedure has more satisfactory ruggedness (Group XI) compared to the other extraction conditions. Efficiency of extraction solvent stored at ambient temperature were 79%, 80% and 74%, respectively (see raw data at Dataset S4. Robustness-ruggedness data of supplementary files), for freshly prepared, one- and three-month-old 80% acetonitrile:water (80:20, *v*/*v*). The HorRat value associated with these data was 0.22, suggesting minimal variability indicating stability of the solvent as an extraction solvent for AFB1 at ambient temperature for three months and satisfactory method ruggedness.

These data, taken together, suggest that the wet milling procedure (slurry method associated with final dilution factor of 1247) had satisfactory robustness as demonstrated by suitable repeatability of extraction efficiency, while stability of extraction solvent in storage at ambient temperature for three months and good within-laboratory reproducibility indicated its acceptable ruggedness [52,54,76]. Good robustness and ruggedness indicate satisfactory stability of our improved aflatoxin test procedure.

#### 2.3.5. Evaluation of the Improved Aflatoxin Test Procedure

The number of replicates of individual samples processed to achieve acceptable level of inter-assay precision (i.e., HorRat ≤ 1) during analysis of 251 field-collected feed samples (routine analysis) is shown in Table 7. A left-skewed one-tailed distribution was observed, with the majority of the samples (75%) adequately analyzed using paired test portions (recommended minimum number) with a further 20% requiring a third run, all these totaling to 95% of the samples. This indicates reduced cost due to sample re-testing in terms of resources and time, decreasing sample analysis turnaround time. Table 8 compares the characteristics of the novel aflatoxin testing procedure with those of other aflatoxin testing protocols recently used to collect aflatoxin residues data in chicken feed. Due to large uncertainty associated with estimation of dietary aflatoxin, FAO [59] has a sampling tool to guide workers on the size of laboratory and test portion samples. For granular products such as finished commercial animal feed, an aggregate sample of more than 2 kg, laboratory sample of not less than 2 kg and test portion sample of at least 50 g (2 × 25 g) have to be processed to reduce results variability. Our modified aflatoxin test adheres to these criteria, while the other published methods fail to meet the appropriate aggregate sample, laboratory sample and/or test portion size (Table 8). Additionally, all of the published methods employed dry milling method for sample homogenization. Because solid–liquid extraction procedures are expensive, workers are tempted to use small test portion samples (which increase inherent variation). Our improved test incorporates a wet milling step (water slurring) in the extraction procedure. This allows processing of the recommended size of the test portion sample of 50 g. Since wet milling is far more effective in sample homogenizing compared to dry milling, a small aliquot of homogenized slurry can be analyzed with minimal inherent variability [35,39,43,61]. For routine analysis, a minimum of two replicates was performed to achieve the required level of inter-assay precision of HorRat ≤ 1 and replicates were increased until this was accomplished (Section 4.2.8).

## 3. Conclusions

The in-house validation data presented here show that the improved aflatoxin test procedure is suitable for estimation of aflatoxin contamination levels in chicken feed. The optimal aflatoxin B1 extraction conditions are wet milling (water slurring 25 g feed in 37.5 mL water), solid–liquid extraction 1.3 g slurry (0.52 matrix: 0.78 water, *w*/*w*) with acetonitrile:water (80:20, *v*/*v*) at dilution rate of slurry/acetonitrile solvent (1:66.5, *w*/*v*) followed by extraction back to aqueous phase at dilution rate of extract/phosphate buffered saline tween 20 (1:6.5, *v*/*v*). The improved aflatoxin test procedure is an accurate, precise, stable, reliable and cost-effective tool (in terms of time and resources) for surveillance of dietary aflatoxin. The improved aflatoxin test procedure described herein is especially suitable for laboratories that may not have access to automated sample-splitting equipment.

## 4. Materials and Methods

### 4.1. Description of Improved Aflatoxin Test Procedure

The improved aflatoxin test procedure for animal feed was designed using FAO mycotoxin sampling tool [40,77] with modification. Briefly, sample selection made from a stationary lot to get a representative aggregate sample (primary sampling) was representatively mass reduced to a 2 kg laboratory sample (secondary sampling) and then two 25 g test samples (tertiary sampling) selected from 2 kg laboratory sample. The test samples were then homogenized by slurring in water, a 1.3 g analytical portion was removed from the analytical sample slurry material (quaternary sampling) and aflatoxin extracted in an organic solvent. The analyte was then extracted back to aqueous phase, phosphate buffered saline tween 20 (PBST) prior to analysis for AFB1 by competitive ELISA.

#### 4.1.1. Sample Selection (Primary Sampling)

The number of bags (sub-lots) sampled was determined as described earlier [49]. Incremental samples (200 g each) were collected by random sampling [49,78] from as many locations of the lot as possible [54], thoroughly mixed to make an aggregate sample (of at least 2 kg). If the number of containers (bags) was ≤10 bags, all were sampled and, if >10 bags, every 4th bag was sampled in every row. All units in the lot were made accessible. To collect incremental samples, a sampling cup was used for open containers (bags) after thorough mixing, while, for closed containers, a bag trier was used. Silica gel packs were added to the aggregate samples, double bagged in brown bags, transported to the laboratory and stored at 4 °C until required [54]. All aggregate samples should first be comminuted prior to mass reduction.

#### 4.1.2. Comminution and Mass Reduction of Samples (Secondary Sampling)

Pellet and crumb aggregate feed samples (varying in size but at least 2 kg) were first comminuted in a laboratory grinder (Grindomix Retsch^®^ Model Gm 200, Hann, Germany) at a rotary speed of 10 × 1000 RPM for 30 s before mass reduction to laboratory samples. This was carried out in 330–350 g aggregate sample portions to protect the grinder from malfunctioning due to overheating. The comminuted aggregate feed samples were then representatively mass-reduced to 2 kg laboratory samples employing coning and quartering technique recommended for sample-splitting of feed samples [49,60] and described earlier [57] but with modification. Briefly, the comminuted aggregate sample was mixed and shoveled into a cone, then flattened by pressing the top without further mixing and dividing the flat circular pile into equal quadrants. Two opposite portions were discarded while the remaining two opposite portions were mixed and shoveled into a cone and the procedure repeated until the material was reduced into four quadrants each of about 500 g. Three quadrants were randomly selected, pooled and the mass topped up to 2 kg laboratory sample on an electronic weighing balance (Mettler PM34, DoltaRange^®^, Zürich, Switzerland)by transferring many small portions randomly picked from the 4th quadrant and double bagged in fresh brown bags. The shoveling and coning process was carried out under a funnel fastened on a tripod stand to ensure uniform distribution of the material.

#### 4.1.3. Preparation of Test Portions (Tertiary Sampling)

The 2 kg laboratory samples were split into four aliquots of about 31 g. Briefly, the 2-kg laboratory samples were representatively mass reduced through five runs of modified coning and quartering method described above until four quadrants each of between 30–35 g were formed. Two 25 g test portions were weighed out from each of opposite portions and the alternate quadrants used for adjusting weights of the selected corresponding test portions if they were below the desired size of 25 g.

#### 4.1.4. Sample Extraction (Quaternary Sampling)

Before blending, dry-run of equipment was carried out to locate particulate contaminants and then cleaned with 70% ethanol. Two (2) 25 g test portions were homogenized by a wet milling step through 2.5-fold dilution proposed earlier for maize samples [59], followed by high speed slurring in water. Briefly, a 25 g test portion was transferred to a kitchen blender cup, 37.5 mL water added (matrix/water ratio of 1:1.5, *w*/*v*) and blended at high speed (Moulinex^®^, Model: Type LM240, Écully, France) for 5 min using a two-step milling protocol (3 min running; 1.5 min pulse; 2 min running) and 1.3 g slurry (0.52 g feed: 0.78 g water) immediately weighed out for extraction and quantification for AFB1. Regular tapping of the blending cup during blending was critical for successful sample homogenization. This reduced splashing of contents on sides of container away from the macerating rotor. Further, the slurry aliquot was weighed out immediately after slurring, directly to the bottom avoiding the neck of the extraction bottle. In each slurry sample, 86.5 mL of acetonitrile (HPLC Grade):water (Milli Q) (80:20, *v*/*v*) was added, contents agitated at 300 rpm for 15 min in orbital shaker-incubator (MRC^®^, Model: Tou-50, Holon, Israel), allowed to settle for 40 min at ambient temperature, supernatant extract decanted and stored at 4 °C.

#### 4.1.5. Quantification of AFB1 in Feed Samples

The extract was prepared and analyzed for AFB1 using AFB1-ELISA low matrix kit (Helica Biosystems Inc.^®^) according to manufacturer’s instructions but with modification. Briefly, 100 µL of the extract, appropriately diluted in phosphate buffered saline containing tween 20 (PBS-T) was loaded on polystyrene ELISA micro-plate with immobilized mouse anti-AFB1 monoclonal antibodies, incubated at ambient temperature for 30 min, contents decanted and plate washed with PBS-T (this was prepared by dissolving contents of PBS-T packet in 1 L distilled water) to remove non-specific reactants, 100 µL of AFB1-horseradish peroxidase conjugate added and incubated again for 30 min, contents decanted and washed with PBS-T to remove non-specific reactants. Substrate-chromogen reagent (100 µL) was added and incubated at ambient temperature for 10 min before stopping the reaction with 100 µL of acid solution and the optical density (ODs), which is inversely proportional to aflatoxin concentration measured at 450 nm on a multi-channel micro-plate reader (BDSL Immunoskan Plus, Finland) inter-faced to a personal computer (Dell^®^, Precision 470, Cherrywood, Ireland) and the OD s recorded using Eiaquik program (© M.C Eisler, 1995). By setting the “zero” standard as 100% binding (B_0_), percent binding for each standard and sample as a percent of the zero binding (% B/B_0_) was calculated. Average absorbance values for each standard and sample extract dilution (B) and that of reagent blank (B_0_) were used to calculate percentage inhibition (B/B_0_%) for each standard and sample dilution, and for construction of 4-parameter calibration curve (log of standard concentration versus logit of B/B_0_%) using programmed template spreadsheets (Microsoft Excel Office 1997–2003). The calibration curve was used to calculate AFB1 concentrations of unknown samples. For the conventional method (Groups VI and VIII), 80% acetonitrile (ACN) extract to PBS-T ratio was 100:900, *v/v*., while for the novel method (Groups II and V) this was modified to 100:650, *v/v* (Table 2).

### 4.2. In-House Validation of Improved Aflatoxin Test Procedure

#### 4.2.1. Materials

Crystalline aflatoxin B1 (Fermentek Ltd., Jerusalem, Israel) was quantified on UV-Vis spectrophotometer (UV-1650 PC, Shimadzu^®^, Kyoto, Japan) by scanning using spectrum mode against methanol blank, absorbance peaks read between 200 and 500 nm as described earlier [79]. Chicken feed specimens (mash form) collected from animal feed retail shops were analyzed for AFB1 using the aforementioned commercial ELISA kit. Specimens with AFB1 levels below the LOD were selected and pooled to make native pseudo blank material [80] for spiking and recovery, and replication studies, and determination of LOD and LOQ. The blank feed material was representatively split to the desired size of test portions employing coning and quartering method recommended for mass reduction of feed samples [49,60] and described earlier [57] with modification described above. AFB1 maize reference (23 ppb) material was acquired from Biosciences eastern and central Africa-International Livestock Research Institute Hub (BecA-ILRI, Nairobi campus) for ruggedness studies.

#### 4.2.2. Study Design

Completely randomized designs for spike and recovery and replication studies and determination of LOD and LOQ are shown in Table 2. There were 11 groups of native pseudo blank feed aliquots (Groupa I–XI) representatively split from the native pseudo blank feed material employing the modified coning and quartering method described above. In addition, 3 groups of acetonitrile extracts of feed aliquots (Groups XII–XIV) were used for LOD and LOQ studies. Five groups were used for spike and recovery studies. First, conventional dry milling (Group I) and our novel wet milling (Group II) procedure were compared (Table 2). Eighteen 25 g aliquots were randomly assigned to a 2 × 3 factorial arrangement i.e., 9 aliquots for each of the two milling methods and three aliquots randomly selected for spiking to achieve three levels of AFB1 (0, 20 or 100 ppb). The EU legal AFB1 residue limits in feed for adult poultry and young animals are 20 and 10 ppb respectively (EU, 2002), but in tropical countries it is common to find feed naturally contaminated with 100 ppb AFB1 and above [13,15,18,19]. Of the 9 aliquots assigned to each group, 3 sets each of 3 aliquots were randomly selected for either of the three AFB1 levels. Secondly, efficiency of various extraction conditions (sample: organic solvent ratio and organic solvent extract: PBST ratio) associated with wet milling procedure were compared. Forty-five 25 g aliquots were randomly assigned to a 3 × 1 factorial arrangement i.e., 15 aliquots for each of the three extraction conditions (Groups III–V) and five aliquots randomly selected for spiking to achieve either of the three levels of AFB1, 0, 20 or 100 ppb. The number of replicates for Groups I and II was therefore 6, and it was 10 for Groups III–V (Table 2). Dry milling procedure involving representative splitting of laboratory sample to obtain test portion, followed by solid–liquid extraction at dilution factor of approximately 5 (Group I) was considered here as the conventional method for recovery studies. The “0” ppb aliquots were used to determine baseline AFB1 levels and were therefore excluded in the sample size (Table 2).

The replication studies used 70 feed aliquots (10 25 g aliquots analyzed whole; 2 25 g aliquots spilt to 20 wet 1.3 g aliquots; 1 25 g aliquot spilt to 10 dry 1.3 g aliquots; and 30 25 g aliquots all spilt to 30 wet 1.3 g aliquots) divided into 6 groups (Table 2). For repeatability, 4 groups (Groups VI–IX) each of 10 feed aliquots were used in a 2 × 3 factorial arrangement to investigate which between dry and wet milling procedures had most suitable intra-laboratory precision for secondary and tertiary sampling stages of sample mass reduction. In addition, intra-laboratory precision for 3 extraction conditions were also investigated. For intermediate precision work, 2 groups (Groups X and XI) each of 15 feed aliquots were used in a 5 × 2 × 3 factorial design to investigate effect of analyst, extraction condition and time on measurement variability. The conventional method (dry milling conditions described in Table 2) for repeatability studies at secondary and tertiary sampling stages are represented by Groups VI and VIII, respectively. For LOD and LOQ determination, field collected feed specimens were screened for AFB1 and one with 8.9 ppb (the lowest level) used as native pseudo blank material. Three groups of extracts derived from this material were used under three extraction conditions. Group XII (dry milling, final dilution factor = 1000) had 4 extracts while Groups XIII (dry milling, final dilution factor = 500) and XIV (wet milling, final dilution factor = 1247) each had 5 extracts. Dry milling procedures (Groups XII and XIII) represent the conventional method (Table 2).

#### 4.2.3. Aflatoxin Spike and Recovery Studies

The solution of AFB1 (Fermentek Ltd., Jerusalem, Israel) in methanol (HPLC Grade) was spiked to achieve 20 or 100 ppb AFB1 level, respectively, in 25 g native pseudo blank feed aliquots, while blank methanol was added to native pseudo blank feed aliquots designated as “0 ppb”.

##### Comparison of Conventional (Dry) and Novel Water Slurry (Wet) Milling Procedure

To a 25 g feed aliquot, 1 mL blank methanol (HPLC Grade), 1 mL of 0.5 or 2.5 µg AFB1/mL solution in methanol (HPLC Grade) was spiked to achieve 0, 20 or 100 ppb AFB1 level, respectively. Group I aliquots were blended in a kitchen blender (Moulinex^®^, Model: Type LM240, Écully, France) at high speed for 1 min, while Group II aliquots were prepared by adding 37.5 mL of water (2.5-fold dilution) and blending as described above (Section 4.1.4), and 2 g slurry immediately weighed out. To each of the 25 g dry and 2 g slurry samples, 133 mL acetonitrile:water (80:20, *v*/*v*) was added and the mixture agitated as described above (Section 4.1.4). Extracts of dry- and wet-blended aliquots were diluted 94.3-fold (final dilution factor = 500) and 7.5-fold (final dilution factor = 1247), respectively, in PBS-T and analyzed for AFB1 by ELISA as described above.

##### Comparison of Different Extraction Conditions Associated with Wet Milling Procedure

The 25 g aliquots were water-slurried and paired 1.3 g slurry analytical samples weighed out as described above (Section 4.1.4). To each of the 1.3 g slurry analytical sample, 130 mL acetonitrile water (80:20, *v/v*) was added for Group III (dilution factor = 100), 65 mL acetonitrile:water (80:20, *v/v*) for Group IV (dilution factor = 50) and 86.5 mL acetonitrile:water (80:20) was added Group V (dilution factor = 66.5), and the mixture was agitated as described in Section 4.1.4. The extracts were diluted 10-fold for Groups III (total dilution factor = 2500) and IV (final dilution factor = 1250) and 7.5-fold for Group V (final dilution factor = 1247) in PBST. The extracts were analyzed for AFB1 by ELISA (Section 4.1.5) and the results of paired test portions for each slurry sample averaged. Mean background level was calculated, subtracted from each of the AFB1 levels of “20 and 100 ppb” replicates and these results expressed as percent recovery. For each group (Groups I–V), mean percent recovery, StDev and RSD of the replicates were calculated. Outlier values in the percent recovery data were identified on Excel 2013 using the following formula:

1st quartile–1.5 IQR ≤ x ≥ 3rd quartile + 1.5 IQR(5)

where IQR is the interquartile range.

Outlier identification criterion also included recommended mean recovery range of 75–125% by European Commission and variance predicted by the modified Horwitz equation [50,51,52,53,63]. Where more than one out of five replicates were identified as outliers, the data were discarded and spiking and recovery procedure repeated [52,53].

#### 4.2.4. Replication Studies

Representativeness of mass reducing methods was investigated as described earlier [48,51,52,63,81].

##### Estimation of Intra-Laboratory Variability (Repeatability)

Ten 25 g test portions were extracted whole while the 11th one (from the same laboratory sample), was water-slurried (Section 4.1.4) and ten 1.3 g slurry aliquots weighed out for extraction. To each of the 25 g of dry (Group VI) and 1.3 g slurry (Group VII) aliquots, 130 mL acetonitrile:water (80:20, *v*/*v*) were added and the mixture agitated (Section 4.1.4), extracts diluted 10-fold in PBST and analyzed for AFB1 by ELISA (Section 4.1.5) to compare variance associated with the dry and wet milling methods at secondary sampling level. Intra-laboratory precision of two procedures was further investigated at tertiary sampling stage. Feed material (50 g) was representatively split into four equal portions by coning and quartering method (Section 4.1.2) and two opposite quadrants pooled to make two equal portions each weighing 25 g. One portion was further split by two runs of 16 aliquots each weighing approximately 1.56 g from which 10 were randomly selected, dry 1.3 g analytical samples weighed (Group VIII) while the other 25 g portion was water slurried and ten 1.3 g slurry aliquots weighed (Group IX). Both dry and slurry samples were extracted and analyzed for AFB1 as described above.

##### Estimation of Intermediate Variability Associated with Wet Milling Procedure

Intermediate precision of the wet milling (slurry) method was also investigated. Five analysts were first trained, each randomly assigned three 25 g native pseudo blank feed aliquots, and each prepared a water slurry daily from one aliquot and weighed out one 1.3 g analytical sample as described above on three consecutive days. Each analytical sample was diluted 100-fold in acetonitrile:water (80:20, *v*/*v*), mixture agitated (Section 4.1.4), extracts diluted 10-fold in PBST and analyzed for AFB1 by ELISA as described above (Group X). This was repeated with another set of fifteen 25 g test portions, but each slurry aliquot was diluted 66.5-fold in extraction solvent, agitated, diluted 7.5-fold in PBST and analyzed for AFB1 (Group XI).

#### 4.2.5. Determination of Limits of Detection (LOD) and Quantification (LOQ)

To counteract matrix effect of chicken feed, we used blank measurement approach to determine LOD and LOQ [81,82]. Briefly a blank specimen was quantified for AFB1 in five measurements and mean OD value and standard deviation obtained. From this, an OD value of mean OD minus 2 standard deviations and mean OD minus 5 standard deviations were calculated to get two OD values which were extrapolated from the standard curve to get LOD and LOQ respectively. Details are given below. Two acetonitrile extracts of the blank specimen prepared by conventional dry milling procedure were pooled, then diluted 100-fold in PBST to attain baseline level of analyte signal and used for LOD and LOQ determination at two assay conditions. Six 100 µL extract aliquots were diluted 100-fold in PBST (Group XII), while the other five 100 µL aliquots were first diluted 5-fold in acetonitrile:water (80:20, *v*/*v*) and then 10-fold in PBS-T (for Group XIII). All the aliquots were further diluted 10-fold in PBST to attain final dilution factor of 1000 for Group XII and 500 for Group XIII for dry milling procedure, before analysis by ELISA. For wet milling procedure, five 25 g fresh feed aliquots were separately water slurried and blended, two 1.3 g analytical portions from each water slurry, weighed and diluted 66.5-fold in acetonitrile:water (80:20, *v*/*v*), contents agitated and extract harvested (Section 4.1.4). The extract was further diluted 66.5-fold in PBS-T to attain baseline level of analyte signal, then diluted 7.5-fold in PBS-T and assayed by ELISA to give a final dilution factor of 1247 (Group XIV) for wet milling procedure. The limit values were calculated from mean absorbance of the 0-standard, B_0_ [63] minus 2-fold (for LOD) and 5-fold (for LOQ) the StDev of absorbance [81] of replicate wells of the blank samples analyzed by ELISA. The means of the concentrations corresponding to the %B/B_0_ value of B_0_ minus 2-fold, and 5-fold the standard deviation generated from the calibration curve was taken as the lowest detectable (LOD) and quantifiable (LOQ) concentration of AFB1.

#### 4.2.6. Linearity Studies

Using pure crystalline AFB1 (Fermentek Ltd., Jerusalem, Israel), 0.5, 0.4, 0.2, 0.1, 0.05 and 0.02 ng/mL AFB1 standards in 80% acetonitrile (HPLC Grade):water (Milli Q) (80:20):PBS-T mixture (10:65) were prepared, analyzed by AFB1-ELISA and the optical density (ODs) of duplicate wells read, as described in Section 4.1.5.

#### 4.2.7. Robustness and Ruggedness Studies

We tested stability of our modified aflatoxin test procedure by examining influence of external factors on its most vulnerable performance parameters [51], precision and accuracy. Intra-laboratory repeatability was used to measure robustness while relatively long-term parameters such as intra-laboratory reproducibility and effect of storage time on extraction efficiency of extraction solvent measured of ruggedness. Determination of robustness was carried out by calculation of repeatability of surrogate recovery (Section 4.2.3). For ruggedness, intra-laboratory reproducibility (effects of day of analysis and analyst) was calculated (Section 4.2.4). Further, the effect of storage on extraction efficiency of organic solvent was investigated. Fresh acetonitrile:water (80:20, *v*/*v*) extraction solvent was prepared and divided into three batches; 1^st^ batch was used immediately, while 2nd and 3rd batches were stored at ambient temperature (minimum = 9.4 ± 1.7 °C; maximum = 25.1 ± 2.9 °C) for one and three months, respectively, prior to use. One gram of 23 ppb maize reference specimen was extracted with 5 mL solvent by agitating and extract harvested (Section 4.1.4), analyzed for AFB1 (Section 4.1.5) and percent recoveries calculated.

#### 4.2.8. Evaluation of the Improved Aflatoxin Test Procedure

In total, 251 chicken feed samples were collected in the field (one sample per lot) as described in Section 4.1.1, prepared and analyzed. Using a programmed spreadsheet (Microsoft Excel Office), minimum requirement criterion for variability associated with sample preparation and analysis was incorporated as a quality control measure. Inter-assay variance associated with sample analysis was evaluated as described earlier [56]. Observed inter-sample variance, observed relative standard deviation (RSD) associated with measurement of paired test portions of each laboratory sample was compared with the predicted relative standard deviation (PRSD) to give a Horwitz Ratio (HorRat) value; thus, HorRat value = RSD_O/_RSD_p_, where RSD_O_ and RSD_p_ are the observed RSD and PRSD, respectively. PRSD was calculated from the modified Horwitz equation which gives RSD as a function of analyte concentration [50,51,52,53,54]: RSD < 2^(1−0.5logC)^ × 0.67, where C is the AFB1 concentration. For samples with HorRat values above 1 and AFB1 levels exceeding the regulatory limit for feed for animal feed (20 ppb), fresh test portions were re-tested until HorRat value of ≤1 was achieved [56]. However, all results from the same sample were averaged to give final mean result and none were discarded. Replicates per sample were recorded and the percent of samples analyzed using 2–6 replicates computed.

### 4.3. Statistical Analysis

Descriptive and inferential data analyses were done on statistical computer program (IBM SPSS Statistics 20). For spiking and recovery data, bias was calculated accordingly: %bias = mean% recovery-100. AFB1 recovery of the conventional dry milling procedure and the novel slurry method were analyzed by comparing means of Groups I and II employing independent *t*-test. Variability expressed as variance, standard deviation and coefficient of variation (relative standard deviation) for Groups I and II were computed and compared. Means of all wet milling procedures recovery data (Groups III–V) were compared employing Welch ANOVA to determine between and within the group variation. Games–Howell test (a post-hoc test) incorporated for multiple comparison of the groups. For spiking and recovery studies, RSD (or CV) effect were determined accordingly,

E_CV_ = G_CV_ − M_CV_ (6)

where E_CV_ = CV effect, G_CV_ = Group CV and M_CV_ = Grand mean CV

The extraction methods ranked according to CV effect values. HorRat values and ORSD/PRSD were used in replication studies to rank sample splitting procedures. For repeatability data, PRSD values were calculated from modified Horwitz equation of PRSD < 2^(1−0.5logC)^ × 0.67, while, for intermediate precision (within-laboratory reproducibility), PRSD was calculated from the unmodified Horwitz equation: PRSD < 2^(1−0.5logC)^, where C is the concentration of the analyte. The intermediate precision data (from replication studies) were further subjected to one-way ANOVA to determine whether the day of analysis and the analyst affected the data. For LOD and LOQ data, outlier OD values were identified using the unmodified Horwitz equation: RSD < 2^(1−0.5logC)^ where, C is the aflatoxin concentration. OD values above B_0_ were also treated as outliers. The HorRat value associated with efficiency of extraction solvent of different shelf ages was used to evaluate variability due to solvent storage time.

## Figures and Tables

**Table 1:** Curve fitting characteristics for the AFB1-ELISA generated by AFB1 standards in modified acetonitrile extract solvent: phosphate buffered saline-20 mixture (100:650, *v*/*v*) showing both values of the parameters (a–d) and linearity-associated coefficients (r and r^2^).

Parameters Values of 4-Parameter Logistic Curve					
Maximum signal intensity (a)	Slope at inflection point (b)	Concentration at inflection point ((50% B/B_0_), IC_50_ (c)	Minimum signal intensity (d)	Coefficients of correlation (r)	Coefficients of determination (r^2^)
98.9	1.58	0.072 ng/mL	5.6	0.998	0.997

**Table 2:** Experimental design for recovery, replication and limits of detection (LOD) and quantification (LOQ) determination studies. Spike and recovery studies utilized surrogate aflatoxin (Groups I–V), whereas replication, LOD and LOQ studies used native aflatoxin (Groups VI–XIV) in feed aliquots.

Feed Aliquot Group	Extraction Conditions ^ε^	Study
I (R = 6) *	(i) dry milling (ii) matrix/ACN ^a^ (25:133, *w*/*v*); (iii) ACN ^a^ extract/PBS-T ^b^ (10.6:989.4, *v*/*v*), (iv) final dilution factor = 500	Spike and recovery (comparison of dry and wet milling)
II (R = 6) *	(i) wet milling @ matrix/water (25:37.5, *w*/*w*); (ii) slurry/ACN ^a^, (2:133, *w*/*v*); (iii) ACN ^a^ extract/PBS-T ^b^ (100:650, *v*/*v*), (iv) final dilution factor = 1247	
III (R = 10) *	(i) wet milling @ matrix/water (25:37.5, *w*/*w*); (ii) slurry/ACN ^a^, (1.3:130, *w*/*v*); (iii) ACN ^a^ extract/PBS-T ^b^ (100:900, *v*/*v*), (iv) final dilution factor = 2500	Spike and recovery studies (comparison of three extraction conditions used in wet milling)
IV (R = 10) *	(i) wet milling @ matrix/water (25:37.5, *w*/*w*); (ii) slurry/ACN ^a^, (1.3:65, *w*/*v*); (iii) ACN ^a^ extract/PBS-T ^b^ (100:900, *v*/*v*), (iv) final dilution factor = 1250	
V (R = 10) *	(i) wet milling @ matrix/water (25:37.5, *w*/*w*); (ii) slurry/ACN ^a^, (1.3:86.5, *w*/*v*); (iii) ACN ^a^ extract/PBS-T ^b^ (100:650, *v*/*v*), (iv) final dilution factor = 1247	
VI (R = 10)	(i) dry milling (ii) matrix/ACN ^a^ (25:130, *w*/*v*); (iii) ACN ^a^ extract/PBS-T ^b^ (100:900, *v*/*v*), (iv) final dilution factor = 52	Intra-laboratory variability (Repeatability)
VII (R = 10)	(i) wet milling @ matrix/water (25:37.5, *w*/*w*); (ii) slurry/ACN ^a^, (1.3:130, *w*/*v*); (iii) ACN ^a^ extract/PBS-T ^b^ (100:900, *v*/*v*), (iv) final dilution factor = 2500	
VIII (R = 10)	(i) dry milling (ii) matrix/ACN ^a^ (1.3:130, *w*/*v*); (iii) ACN ^a^ extract/PBS-T ^b^ (100:900, *v*/*v*), (iv) final dilution factor = 1000	
IX (R = 10)	(i) wet milling @ matrix/water (25:37.5, *w*/*w*); (ii) slurry/ACN ^a^, (1.3:130, *w*/*v*); (iii) ACN ^a^ extract/PBS-T ^b^ (100:900, *v*/*v*) (iv) final dilution factor = 2500	
X (R = 15)	(i) wet milling @ matrix/water (25:37.5, *w*/*w*); (ii) slurry n/ACN ^a^, (1.3:130, *w*/*v*); (iii) ACN ^a^ extract: PBS-T ^b^ (100:900, *v*/*v*); (iv) final dilution factor = 2500	Intermediate variability (within-laboratory reproducibility)
XI (R = 15)	(i) wet milling @ matrix/water (25:37.5, *w*/*w*); (ii) slurry/ACN ^a^, (1.3:86.5, *w*/*v*); (iii) ACN ^a^ extract/PBS-T ^b^ (100:650, *v*/*v*) (iv) Final dilution factor = 1247	
XII (R = 4)	(i) dry milling (ii) ACN ^a^ extract/PBS-T ^b^ (1:1000, *v*/*v*) (iii) final dilution factor = 1000	Determination of LOD and LOQ
XIII (R = 5)	(i) dry milling ACN ^a^ extract/ACN ^a^/PBS-T ^b^ (1:5:100, *v*/*v*/*v*) (ii) final dilution factor = 500	
XIV (R = 5)	(i) wet milling (ii) ACN ^a^ extract/PBS-T ^b^ (1:1247, *v*/*v*) (iii) final dilution factor = 1247	

**^ε^** Extraction conditions here mean sample homogenization, solid–liquid and liquid–liquid extraction solvent mixture components with Roman numbers (i–iii) standing for specific conditions whose details can be found in Section 4.2.2, Section 4.2.3 and Section 4.2.4; R is n = number of replicates; * number of “0” ppb feed aliquots not included in *n.*; ^a^ acetonitrile:water (80:20, *v*/*v*); ^b^ phosphate buffered saline tween 20.

**Table 3:** Extraction efficiency (percent recovery), bias and measurement precision of surrogate AFB1 content in chicken feed associated with dry and wet sample homogenization procedures at various extraction conditions (dilution factors) and ranked using HorRat* value effect.

Milling Method	Group	Aflatoxin Extraction Conditions						* Mean AFB1 % Recovery	% Bias	Measurement Variability			
		Expected Concentration	Size of Analytical Sample	DF of Water Slurry	DF in ACN *	DF in PBS-T ^b^	Final DF			StDev.	CV	CV Effect	HorRat ** Value
Dry	I (R = 6)	20 ppb (R = 3)	25 g	-	5.3	94.3	500	57		±6	10.9		0.57
		100 ppb (R = 3)						103		±13	12.4		0.82
		All replicates						80 ^a^	−20	±26	33.1	9.5 ^R4^	0.57–0.82
Wet	II (R = 6)	20 ppb (R = 3)	2 g	2.5	66.5	7.5	1247	119		±25	21.2		0.95
		100 ppb (R = 3)						123		±9	7.4		0.8
		All replicates						121 ^a^	21	±17	14.1 ^h^	−9.5 ^R2^	0.8–0.95
Wet	III (R = 10)	20 ppb (R = 5)	1.3 g	2.5	100	10	2500	145		±19	12.8		0.58
		100 ppb (R = 5)						112		±9	17.4		0.44
		All replicates						129 ^b,c^	29	±22	17.2	−3.8 ^R3^	0.44–0.58
	IV (R = 10)	20 ppb (R = 5)	1.3 g	2.5	50	10	1250	49		±12	23.2		1.04
		100 ppb (R = 5)						85		±15	18.1		1.04
		All replicates						67 ^b^	−33	±23	34.2	13.3 ^R5^	1.04
	V (R = 10)	20 ppb (R = 5)	1.3 g	2.5	66.5	7.5	1247	80		±13	15.8		0.71
		100 ppb (R = 5)						80		±6	6.9		0.39
		All replicates						80 ^c^	−20	±9	11.5	−9.5 ^R1^	0.39–0.71
Groups II and V replicates analyzed together (R = 16)		20 ppb (R = 8)	-	2.5	66.5	7.5	1247	94		±26	27.6		1.24
		100 ppb (R = 8)						96		±23	24.1		1.38
		All replicates						95	−5	±24	25.0		1.24–1.38

** Observed residue standard deviation/predicted residue standard deviation; R is n = number of replicates; * acetonitrile:water (80:20, *v*/*v*), ^a^ figures marked with this superscript were statistically compared by independent *t*-test; ^b^ phosphate buffered saline tween 20; DF, Dilution factor; StDev, Standard deviation; CV, Coefficient of variation (relative standard deviation); ^b,c^ figures marked with the same superscript were statistically compared by Welch’s ANOVA (Games-Howel post-hoc test); ^R1−5^ method ranking based on CV effect with descending order of preference; * 75–125% mean recovery is the accepted recovery range for trace AFB1 levels [52].

**Table 4:** Within laboratory repeatability and reproducibility associated with measurement of native aflatoxin content in chicken feed at secondary and tertiary sampling stages after sample homogenization employing by either dry or wet (water slurring) milling techniques. Variability values were determined under different extraction conditions and ranked using HorRat * value effect.

Sampling Stage	Intra-Laboratory Assay Precision (Within-Laboratory Repeatability)						Intermediate Precision (Within-Laboratory Reproducibility)					
	Group Number/ Mass Reduction Method	Milling Method and Size of Analytical Sample	EC	AFB1 Level (ppb) Mean ± Sd.	Variability		Group Number/ Mass Reduction Method	Milling Method and Size of Analytical Sample	EC	AFB1 Level (ppb) Mean ± Sd	Variability	
					RSD_r_	HorRat ValueEffect					RSD_R_	HorRat Value Effect
Secondary	Group VI Coning and quartering (R = 10)	Dry 25 g	1	15 ± 1	6.5	0.3 ^H1^	-					
Tertiary	Group VII Water slurry (R = 10)	Wet 1.3 g	2	68 ± 17	24.5	1.5 ^H5^	Group X Water slurry (R = 15)	Wet 1.3 g	2	69 ± 21	30.3	1.3 ^H4^
Tertiary	Group VIII Coning and quartering (R = 10)	Dry 1.3 g	3	73 ± 67	91.5	5.8 ^H6^	Group XI Water slurry (R = 15)	Wet 1.3 g	4	19 ± 5	26.9	0.9 ^H2^
Tertiary	Group IX Water slurry (R = 10)	Wet 1.3 g	2	50 ± 10	20.6	1.2 ^H3^	-					

* Observed residue standard deviation/predicted residue standard deviation; R is n = number of replicates; EC (Extraction conditions) 1 enumerated as i–iv: (i) dry milling (ii) matrix/ 80% acetonitrile (25:130, *w*/*v*); (iii) 80% acetonitrile extract/ phosphate buffered saline tween 20 (100:900, *v*/*v*); (iv) final dilution factor = 52: EC 2 enumerated as i–iv: (i) wet milling with matrix/water (25:37.5, *w*/*w*); (ii) slurry/80% acetonitrile (1.3:130, *w*/*v*); (iii) 80% acetonitrile extract/ phosphate buffered saline tween 20 (100:900, *v*/*v*); (iv) final dilution factor = 2500: EC 3 enumerated as i-iv: (i) dry milling; (ii) matrix/ 80% acetonitrile (1.3:130, *w*/*v*); (iii) 80% acetonitrile extract/ phosphate buffered saline tween 20 (100:900, *v*/*v*); (iv) final dilution factor = 1000: EC 4 enumerated as i–iv: (i) wet milling with matrix/water (25:37.5, *w*/*w*); (ii) slurry/80% acetonitrile (1.3:86.5, *w*/*v*); (iii) 80% acetonitrile extract/ phosphate buffered saline tween 20 (100:650, *v*/*v*); (iv) final dilution factor = 1247: Sd, Standard deviation; RSD**_r_**, within-laboratory repeatability relative standard deviation; RSD**_R_**, within-laboratory reproducibility relative standard deviation; ^H1–6^ method ranking based on HorRat value effect with descending order of preference.

**Table 5:** Limits of detection (LOD) and quantification (LOQ) values associated with dry and wet (water slurring) sample homogenization procedures for chicken feed at different extraction conditions (dilution factors). The LOD and LOQ values were derived from signal and noise of baseline native aflatoxin content expressed as absorbance.

Baseline Response (OD) Statistics	Limits of Detection and Quantification in µg AFB1 Per kg of Chicken Feed		
	Dry Milling		Wet Milling
	Group XII (R = 4) (Final Dilution Factor = 1000)	Group XIII (R = 5) (Final Dilution Factor = 500)	Group XIV (R = 5) (Final Dilution Factor = 1247)
Mean-2StDev (Limit of detection)	16.3	10.6	7.5
Mean-5StDev (Limit of quantification)	31	22.3	16
Baseline response (OD) statistics	Magnitude of signal, noise, their ratio and precision (absorbance)		
	Group XII	Group XIII	Group XIV
B_0_	1.773	1.752	1.871
StDev (blank)	0.0399	0.0485	0.0339
Signal/noise ratio	44.44	36.12	55.19
Coefficient of variance	2.34	2.99	1.97

R is n = number of replicates; OD, optical density; B_0,_ average OD of the 0 standard; Mean, average of B_0_ OD values; StDev, standard deviation of OD values of blank feed material.

**Table 6:** Linearity of aflatoxin B1 standard solutions in modified acetonitrile extract solvent: phosphate buffered saline tween 20 mixture (100:650, *v*/*v*). The values are mean of duplicate analysis.

	Concentration and Optical Density of AFB1 Standard Solutions					
	Solution 1	Solution 2	Solution 3	Solution 4	Solution 5	Solution 6
**Spiked AFB1 level (ng/mL)**	0.02	0.05	0.1	0.2	0.4	0.5
**Measured level (ng/mL)**	0.03	0.06	0.12	0.28	0.36	>0.4
**Measured optical density**	1.429	1.106	0.663	0.297	0.226	0.199

**Table 7:** Number of replicates (25 g test portions) required to attain acceptable level of precision during routine analysis of aflatoxin B1 in chicken feed employing the improved test procedure. Percentage of samples analyzed is included in brackets. Paired test portion (duplicate analysis) is the minimum number of replicates recommended by FAO Mycotoxin Sampling tool.

	Number of Replicates Required to Attain Acceptable Intra-Assay Precision				
	Two Replicates Required	Three Replicates Required	Four Replicates Required	Five Replicates Required	Six Replicates Required
Number and percentage of samples analyzed	188 (74.9%)	51 (20.3%)	8 (3.2%)	2 (0.8%)	2 (0.8%)

**Table 8:** Sample homogenization techniques, size of aggregate, laboratory and test portion samples of various aflatoxin analysis protocols compared to the modified (novel) test procedure. This highlights compliance of our modified to FAO criteria for aflatoxin analysis.

Study Reference	Characteristics of Aflatoxin Test Procedures				
	Homogenization Method	Aggregate Sample (kg)	Laboratory Sample (kg)	Size of Test Portion	Analytical Method
[15]	Dry milling	4	Not given	5 g	LC-MS/MS
[16]	Dry milling	1–2	1	5 g	ELISA
[19]	Dry milling	1	1	5 g	LC-MS/MS
[32]	Dry milling	Not given	Not given	Not given	HPLC/ELISA
[18]	Dry milling	Not given	Not given	50 g	VICAM Fluorimeter
[13]	Not given	Not given	Not given	Not given	Not given
[14]	Not given	1	Not given	Not given	TLC
Novel method	Wet milling (water slurring)	>2	2	at least 25 g × 2	ELISA or any other quantification method

## Data Availability

The data presented in this study are available as supplementary material (Dataset S1. Aflatoxin recovery data; Dataset S2. Precision data; Dataset S3. LOD and LOQ data; Dataset S4. Robustness-ruggedness data).

## Supplementary Materials

The following are available online at https://www.mdpi.com/2072-6651/13/3/216/s1, Dataset S1. Aflatoxin recovery data, Dataset S2. Precision data, Dataset S3. LOD and LOQ data, Dataset S4. Robustness-ruggedness data.
